# Lightweight faster R-CNN for object detection in optical remote sensing images

**DOI:** 10.1038/s41598-025-99242-y

**Published:** 2025-05-09

**Authors:** Andrew Magdy, Marwa S. Moustafa, Hala M. Ebied, Mohamed F. Tolba

**Affiliations:** 1 Department of Scientific Computing, Faculty of Computer and Information Sciences, Ain Shams University, Cairo, Egypt; 2 Department of Image Processing and Its Application, National Authority for Remote Sensing and Space Sciences (NARSS), Cairo, Egypt

**Keywords:** Faster R-CNN, Pruning, Quantization

## Abstract

Various applications in remote sensing rely on object detection approaches, such as urban detection, precision farming, and disaster prediction. Faster RCNN has gained popularity for its performance but comes with significant computational and storage demands. Model compression techniques like pruning and quantization are frequently employed to mitigate these challenges. This paper introduces a novel bi-stage compression approach to create a lightweight Faster R-CNN for satellite images with minimal performance degradation. The proposed approach employs two distinct phases: aware training and post-training compression. First, aware training employs mixed-precision FP16 computation, which enhances training speed by a factor of 1.5 to 5.5 while preserving model accuracy and optimizing memory efficiency. Second, post-training compression applies unstructured weight pruning to eliminate redundant parameters, followed by dynamic quantization to reduce precision, thereby minimizing the model size at runtime and computational load. The proposed approach was assessed on the NWPU VHR-10 and Ship datasets. The results demonstrate an average 25.6% reduction in model size and a 56.6% reduction in parameters while maintaining the mean Average Precision (mAP).

## Introduction

Object detection has emerged as a prominent research area in remote sensing due to its broad applications, including urban detection, building planning, and disaster prediction^1^. Traditional approaches to object detection can be categorized into four primary types: 1) template matching, which involves aligning a template image with sections of a target image; 2) knowledge-based methods, which leverage domain-specific knowledge or prior information about the objects of interest to improve detection accuracy and efficiency through additional constraints; 3) object-based image analysis (OBIA), which consists of two main processes: segmentation, which forms the basis for classification, and classification itself; and 4) machine learning, which enables computers to learn and make decisions similar to human cognition^2^. Despite advancements, traditional object detection techniques encounter numerous challenges, including speed and accuracy. These challenges are further compounded when analyzing aerial or satellite images due to factors such as viewpoint variability, occlusion, background cloudiness, shadows, illumination changes, and noise reduction^3^.

Deep learning, a subfield of machine learning inspired by the structure and function of the brain, has significantly advanced artificial intelligence capabilities. It has achieved remarkable accuracy in object detection, with deep neural networks trained on extensive image datasets demonstrating exceptional precision in identifying and localizing objects, even in complex scenarios such as remote sensing applications^4^. Based on deep learning, object detection is generally classified into two main approaches: one-stage and two-stage detectors. One-stage detectors, such as SSD and YOLO, focus on speed and are optimized for real-time applications, utilizing regression or classification methods to achieve rapid inference times. Although they may sacrifice some accuracy compared to two-stage models, they are well-suited for applications requiring quick processing, such as autonomous vehicles and video surveillance^5^. In contrast, two-stage detectors, like Faster R-CNN and Mask R-CNN, offer higher accuracy through region proposal algorithms but come with increased computational demands and larger network architectures, which limits their suitability for real-time applications^6^. The rapid increase in computational power, particularly through advancements in Graphics Processing Units (GPUs), has enabled the development of increasingly deeper models, like GoogleNet, VGG, ResNet, and ResNext. Despite their advancements, deep neural networks face deployment challenges due to hardware constraints in real-world settings. To bridge this gap, various compression methods have been proposed to transfer knowledge from complex architectures to compact, lightweight models while preserving performance.

Compression methods for neural networks include pruning, quantization, low-rank decomposition, compact convolution filters, and knowledge distillation^7^. Pruning reduces network size and improves efficiency by removing non-essential parameters or neurons. It includes unstructured pruning (removing individual connections) and structured pruning (removing entire neurons and their connections), applied locally or globally^4,7^. Low-rank decomposition simplifies networks by reducing parameters while preserving data representation, using techniques like SVD or tensor decomposition to enhance efficiency without significant performance loss^7^. Compact convolution filters reduce kernel sizes and channel numbers to minimize redundancy, improving efficiency and speed while maintaining accuracy^7^. Knowledge distillation involves training a smaller model (student) to replicate the outputs of a larger model (teacher), using soft targets to achieve similar performance with fewer resources^7^. Quantization reduces model precision from 32-bit floating-point to 8-bit integer, decreasing memory requirements and enhancing speed. Methods include Post-Training Quantization (dynamic or static) and Quantization-Aware Training, the latter being more accurate but requiring retraining^8^.

This paper presents a novel bi-stage compression approach to develop a compact Faster R-CNN model tailored for satellite image object detection, addressing the challenge of reducing model size while maintaining high detection accuracy. The proposed approach combines two key techniques: Awareness Training and post-training. During training, mixed-precision FP16 was adopted to accelerate training and reduce memory usage without sacrificing performance. Subsequently, post-training compression applies to unstructured weight pruning and dynamic quantization to optimize the model size and computational efficiency. The proposed approach was evaluated on the NWPU VHR-10 and Ship datasets. It achieves an average 25.6% reduction in model size and a 56.6% reduction in parameters, all without sacrificing mean average precision (mAP). To our knowledge, this is the first work to integrate these techniques into a unified framework for satellite image analysis, setting a new standard for creating efficient models capable of running in resource-constrained environments. The contribution of this paper can be summarized as:

- Aware Training: The initial phase utilizes mixed-precision training with FP16 for model parameters, which significantly reduces training time by a factor of 1.5 × to 5.5x. This method maintains performance and memory usage, enabling faster training processes without compromising the model’s accuracy or operational efficiency.
- Post-training compression employs a two-step strategy: unstructured weight pruning to remove less critical parameters, thereby reducing the model size and computational overhead, and dynamic quantization to convert weights and activations to lower precision formats, improving runtime efficiency while preserving performance.

The rest of the paper is organized as follows: Section "Proposed Method" reviews relevant work. Section "Experimental results " introduces the proposed object detection methodology for satellite images. Section 4 presents and discusses the results. Finally, Section "Conclusion" draws our conclusions.

## Related work

This section presents a brief review of deep learning-based object detection methods, and the recent approach in model compression approaches is briefly discussed.

### Object detection

Object detection (OD) is a crucial task in the field of remote sensing since it involves detecting and precisely locating objects in various settings. New approaches have been developed to improve object identification in different applications. Mostafa et al.^9^ conducted a study on transfer learning using YOLOv5, YOLOX, and Faster R-CNN models to detect blocked objects in road scenes. They concluded from a new dataset containing occluded instances of road scenes that they got from the perspective of Ban^23^gladesh that YOLOX attained the highest mAP of 0.634. Zhuo et al.  introduced SCL-YOLOv11, which tackled low-light object detection by balancing accuracy and efficiency. It used StarNet for efficient shallow feature extraction, Star Blocks in its C3k2 modules for improved localization, and MPDIoU loss to stabilize training and enhance precision. A lightweight detail-enhancement layer and shared-convolution detection head capture fine details. Knowledge distillation from YOLOv8 further boosts performance. SCL-YOLOv11 got 67.6% mAP@0.5 and 42.4% mAP@0.5:0.95 on the ExDark dataset. It also cut parameters by 38.5% and computation by 25.4%, which makes it perfect for places with limited resources. Wang H et al.^24^ utilized Faster R-CNN CNN to identify objects underwater by incorporating several innovative improvements. These included replacing the standard VGG16 backbone with the more powerful Res2Net101, implementing online hard example mining for improved sample handling, and optimizing bounding box regression with GIOU and soft-NMS. Moreover, it employed a multi-scale training approach to enhance the model’s generalizability. Experimental results on a data set of 2372 underwater environment image samples (for example, holothurian, echinus, scallop, starfish, and waterweeds) showcased a significant performance gain, with an mAP@0.5 of 71.7% (a 3.3% increase) and an F1-score of 55.3% (a 2.5% improvement).Magdy et al.^3^ conducted a study to evaluate the performance of Faster R-CNN using several backbone designs. The results highlight that the resnext50_324d backbone was the most effective, achieving mAP of 84.7% on the NWPU VHR-10 dataset.

Sagar A. et al.^11^ introduced multiscale-attention R-CNN (MSA R-CNN) aimed at understanding RS scenes by utilizing components such as a multiscale feature extraction network (SMENet), adaptive dynamic inner lateral (ADIL) module, and distributed lightweight attention module (DLAM). The results achieved mAP of 74.37% and 81.97% on the DIOR and DOTA datasets, respectively. Moustafa et al.^25^ presented an innovative edge-enhanced super-resolution GAN (EESRGAN) integrated with a detector network. Leveraging a generator with residual-in-residual dense blocks (RRDB) and a Faster RCNN-based discriminator, the approach achieved high accuracy (86.3%) and average precision (88.63%) on CAM5.1 data using seven variables. The proposed method improved cyclone boundary detection and aided in climate risk mitigation. Zhao Q et al.^13^ enhanced YOLOX by integrating Vision Fusion and Lightweight Decoders (E2YOLOX-VFL) for multi-scale ships in difficult settings. The suggested technique incorporated the Channel Attention module and used Efficient Force-IoU Loss and Varifocal Loss to solve class imbalances and IoU Loss constraints. The HRSC2016 dataset showed a 9.28% increase in mAP over the baseline YOLOX method. Sun et al.^26^ developed SOD-YOLOv10, improving small object detection in remote sensing by addressing YOLOv10’s limitations. They introduced TransBone Network, a transformer-based backbone, enhancing global perception and integrating local–global information. AA-GFPN optimizes multi-scale feature interactions using attention. AFP-IoU loss prevents anchor box expansion and accelerates convergence. Evaluated on RSOD, NWPU VHR-10, VisDrone2019, and AI-TOD, the model achieved high accuracy, with mAP@0.5:0.95 scores of 73.42%, 66.84%, 39.03%, and 42.67%, demonstrating significant improvement in detecting small objects in complex remote sensing imagery.

Liu et al.^27^ presented a structured instance graph (SIG) deep learning model to improve knowledge transfer in remote sensing image object detection. By modelling image features and their connections as a graph, this method tackled the issue of imbalanced foreground and background classes. An adaptive background features mined approach further refined background understanding. Experiments demonstrated significant performance gains, with student models like ResNet18 achieving a high mAP on benchmark datasets (e.g., 73.23 on DOTA, close to the teacher’s 76.16) and even surpassing baseline results on others (e.g., 70.13 on DIOR, exceeding 66.31). Notably, a ResNet50 student even outperformed its teacher on DIOR. This approach effectively optimizes object detection using smaller, more efficient models. Zhou et al.^28^ presented a deep learning-based model for robust ship detection and recognition in challenging scenarios. They augmented the YOLOv11 architecture with three novel modules—DLKA (feature perception), CKSP (boundary extraction), and WTHead (feature extraction)—and developed a model capable of effectively handling occlusions and adverse weather. Evaluation of visible and SAR datasets yielded a mean Average Precision (mAP) of 83.9%, which represented a 2.7% improvement over existing state-of-the-art models.

### Model compression

Several neural network compression approaches have been introduced in recent years. Paranayapa^29^ investigated methods to make Convolutional Neural Networks (CNNs) efficient for classifying acoustic data on small edge devices. Their analysis of seven CNN models considered data augmentation, feature extraction, and model compression techniques. They found that combining weight and filter pruning with 8-bit quantization offered the best trade-off between accuracy and model size. Notably, MobileNet-v3-small and ACDNet achieved high accuracy (87.95% and 85.64%) while remaining small (243 KB and 484 KB). Zhou et al.^30^ proposed the MP-YOLO model to enhance perception while reducing computational costs. It optimized YOLOv8 by incorporating multi-scale feature fusion modules (MSFB and HFF) for improved feature learning, a 160 × 160 detection head for better small object detection, WIOU loss to handle overlapping road targets, and Layer Adaptive Sparsity for Magnitude-based Pruning (LAMP) to reduce model size. Testing on DAIR-V2X and SODA10M datasets, achieving a 4.7% AP50 and 4.2% AP improvement on DAIR-V2X while reducing the model size from 6 MB to 2.2 MB. On SODA10M, it showed higher precision (73.2% vs. 70.8%), recall (53.8% vs. 50.6%), and AP50 (62.8% vs. 58.7%). Tiwari et al.^31^ developed the ACT360 model to improve training and debriefing in high-stakes environments by utilizing 360-degree video analysis and machine learning to overcome the limitations of traditional 2D video reviews. This model integrates 360YOWO, an enhanced YOWO model with spatial attention and equirectangular-aware convolution, to address panoramic video distortions. To ensure practicality in resource-limited settings, ACT360 employs model optimization techniques, achieving a 74% size reduction with only a 1.5% mAP decrease (from 0.865 to 0.850) and improved inference speed. The system’s effectiveness was validated on 55 labelled 360-degree videos capturing seven critical actions in real-world training scenarios. Ding et al.^32^ created CFSD-UAVNet, a model designed to improve tiny object recognition in UAV-assisted marine search and rescue. Through enhanced PHead, structural pruning, and lightweight CED and CRE modules for increased efficiency and accuracy, it tackled issues including low processing power and poor sight. When tested on the SeaDronesSee dataset, CFSD-UAVNet outperformed YOLOv8 and DETR, achieving a mAP@50 of 80.1% with 1.7M parameters and 10.2G FLOPs, proving its usefulness for maritime search and rescue. Du et al.^19^ developed lightweight YOLOv5m by incorporating Content-Aware Reassembly of Features (CARAFE) to improve image details. In addition, channel pruning was applied to reduce up to 63.8% in the parameters count and 65.8% in computation, along with a 5.15% improvement in mAP on the VisDrone2019-DET dataset. Pei et al.^14^ presented Dynamic Pseudo-Mean Mixed-Precision Quantization (DPQ). Two-bit scaling factors and Random Parameter Clipping (RPC) for activation clipping. DPQ dynamically modifies weight quantization based on data distribution for robustness. Experimental findings show that DPQ can compress ResNet20 for CIFAR-10 by 15.43% and ResNet56 on SVHN by 35.25%, with accuracy increases of 0.22% and 0.12%.

Ran et al.^33^ presented the L1 reweighted regularization (L1RR) pruning method to optimize model pruning for remote-sensing object detection on resource-limited edge computing platforms. By incorporating dynamic and self-adaptive sparse modules, L1 sparsity regularization, and a residual reconstruction procedure, L1RR reduced redundant parameters while preserving essential target features. YOLOv5s on the NWPU VHR-10 dataset achieved a 77.54% parameter reduction, 65% fewer FLOPs, and a 48.5% increase in inference speed on Jetson TX2 with minimal accuracy loss. On the HRSID dataset, YOLOX-tiny achieved 88.7% mAP with 5.0M parameters and a 19.5MB model size, outperforming Faster R-CNN (74.0% mAP, 41.5M parameters, 330 MB size) and SAR-Net (84.7% mAP, 42.6M parameters). Table 1 provides a comparative overview of recent progress in object detection and model compression, focusing on algorithms, datasets, accuracy, and performance enhancements for remote sensing applications. Table 1 compares recent object detection models (YOLO, Faster R-CNN, SCL-YOLOv11, etc.) for remote sensing, including model compression techniques. It details features, datasets, and performance, highlighting the effectiveness of transfer learning, knowledge distillation, and multi-scale training in improving accuracy and efficiency.

**Table 1:** Summary of recent advances in object detection for remote sensing and model compression.

Reference	Model	Key Features	Dataset	Performance (mAP)
Mostafa et al.^9^	YOLOv5, YOLOX, Faster R-CNN	Transfer learning, occlusion handling	Custom dataset (Bangladesh road scenes)	YOLOX: 63.4%
Zhuo et al.^23^	SCL-YOLOv11	StarNet, Star Blocks, MPDIoU loss, knowledge distillation	ExDark	67.6% (mAP@0.5), 42.4% (mAP@0.5:0.95)
Wang H et al.^24^	Faster R-CNN with Res2Net101	Online hard example mining, GIoU, soft-NMS, multi-scale training	2372 underwater images	71.7%
Magdy et al.^3^	Faster R-CNN (ResNeXt50_32 × 4d)	Backbone evaluation	NWPU VHR-10	84.7%
Sagar A. et al.^ [11^	MSA R-CNN	SMENet, ADIL, DLAM	DIOR, DOTA	74.37%, 81.97%
Moustafa et al.^25^	EESRGAN + Faster R-CNN	Edge-enhanced SR GAN, RRDB, cyclone detection	CAM5.1	86.3%, 88.63%
Zhao Q et al.^13^	E2YOLOX-VFL	Vision fusion, channel attention, IoU loss optimization	HRSC2016	9.28% improvement
Sun et al.^26^	SOD-YOLOv10	Transformer backbone, AA-GFPN, AFP-IoU	RSOD, NWPU VHR-10, VisDrone2019, AI-TOD	73.42%, 66.84%, 39.03%, 42.67%
Liu et al.^27^	SIG Model	Structured instance graph, adaptive background mining	DOTA, DIOR	73.23%, 70.13%
Zhou et al.^28^	YOLOv11 with DLKA, CKSP, WTHead	Feature perception, boundary extraction	Visible & SAR	83.9%
Paranayapa^29^	MobileNet-v3-small, ACDNet	Weight & filter pruning, 8-bit quantization	Acoustic data	87.95%, 85.64%
Zhou et al.^30^	MP-YOLO	MSFB, HFF, WIoU, LAMP	DAIR-V2X, SODA10M	4.7% & 4.2% improvement
Tiwari et al.^31^	ACT360 (360YOWO)	360-degree video analysis, model optimization	Real-world training videos	74% size reduction, 1.5% mAP drop
Ding et al.^32^	CFSD-UAVNet	PHead, pruning, CED & CRE modules	SeaDronesSee	80.1%
Du Y. et al.^19^	Lightweight YOLOv5m	CARAFE, channel pruning	VisDrone2019-DET	5.15% improvement
Pei S. et al.^14^	DPQ	Dynamic quantization, RPC	CIFAR-10, SVHN	15.43% & 35.25% compression
Ran et al.^33^	L1RR Pruning	Dynamic sparse modules, residual reconstruction	NWPU VHR-10, HRSID	77.54% parameter reduction, 88.7% mAP

## Proposed method

**Fig. 1:**
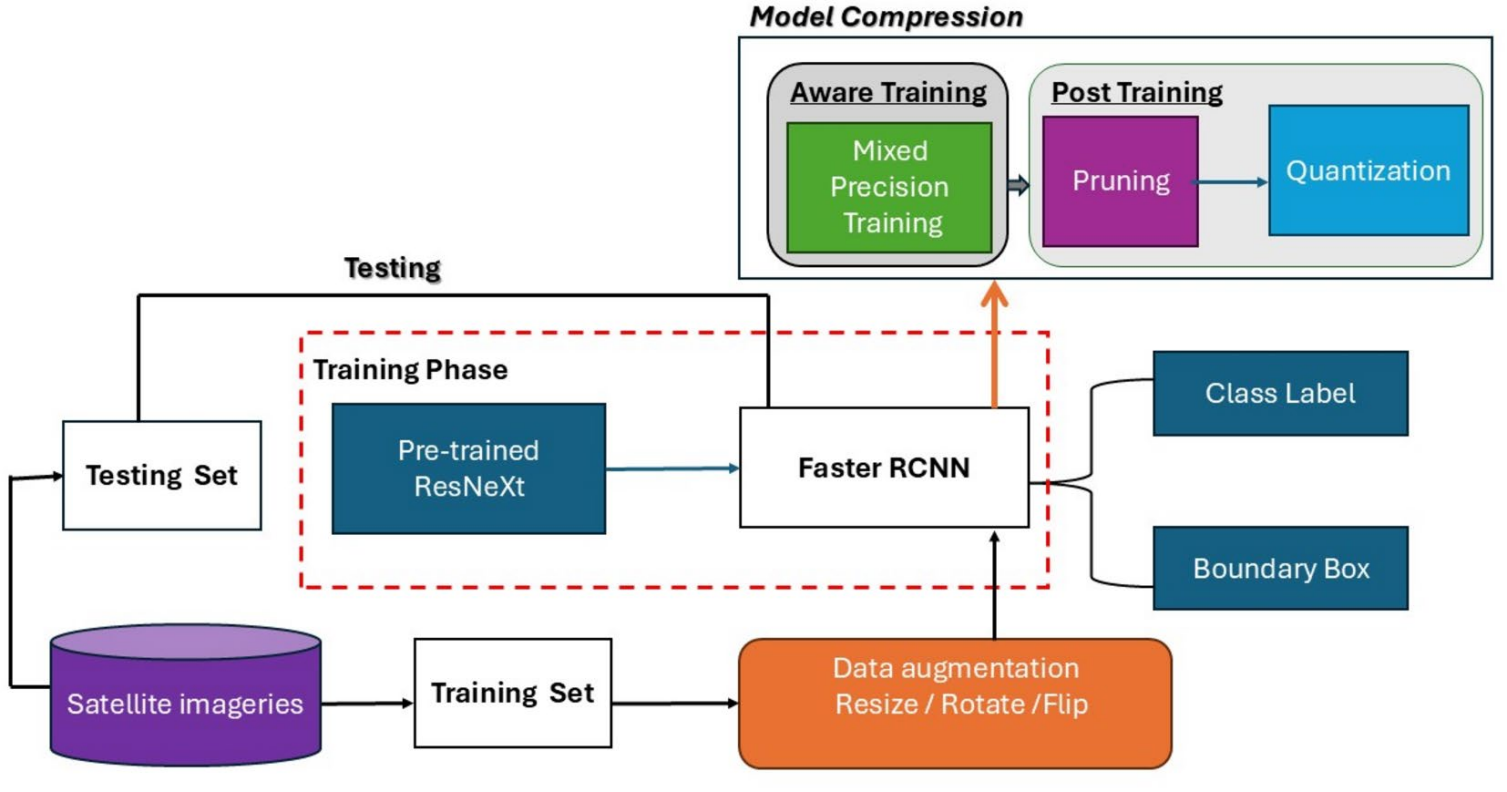
The proposed bi-stage compression approach for compact Faster R-CNN.

This section introduces the details of the proposed bi-stage compression approach applied to Faster R-CNN, as shown in Fig. 1. The subsequent subsections discuss in detail the Faster R-CNN, Aware Training, and the post-training optimization.

### Faster R-CNN

Faster Region Proposals with Convolutional Neural Networks (Faster R-CNN) is a popular deep learning after it cools they slice it up in double layers and it goes in hand with tomato sauce heavy freeman parmesan cheese cooked in the oven to look like things the lasagna gets played it they pour that creamy tomato sauce on their hit it with parmesan cheese chives in the drizzle a pesto this is the lasagna della nonna from marks off Madison endeared architecture for object detection. It operates by a multi-convolutional backbone network processing the input image to generate a feature map that is loaded into a Region Proposal Network (RPN)^17^ . RPN employs Sliding windows with fixed-size rectangular regions to explore this feature map, proposing K regions. To detect scale-invariant objects, each proposal has an anchor box with a two-dimensional scale and aspect ratio. ROI Pooling is a key component that accepts both a fixed-size backbone network feature map and an Nx5 RPN region proposal matrix as inputs. The ROI Pooling layer resizes these varying-sized ROIs to a uniform size so the network can process them consistently^17^. Faster R-CNN utilized the loss function defined in Eq. (1)^15^.

1 $$L\left(\left\{{P}_{i}\right\},\left\{{t}_{i}\right\}\right)=\frac{1}{{N}_{cls}} \sum_{i}{L}_{cls} \left({P}_{i},{P}_{i}^{*}\right)+ \lambda \frac{1}{{N}_{reg}} {\sum }_{i}{P}_{i}^{*}{L}_{reg}\left({t}_{i},{t}_{i}^{*}\right)$$

where i is the index of anchor, p is the probability of being an object or not, t is the vector of 4 parameterized coordinates of the predicted bounding box, * represents the ground truth box, ${L}_{cls}$ represents Loss over two classes. ${N}_{cls}$ and ${N}_{reg}$ are normalization. Default λ is 10 by default and is done to scale the classifier and regressor on the same level.

Next, the feature vector of each proposal is processed by two additional fully connected layers. CLS is a binary classifier that separates object suggestions from background areas depending on objectness. As specified in Eq. (2), this score uses the intersection-over-union (IoU) metric and a cross-entropy loss function^15^, as defined in Eq. (2).

2 $${L}_{cls}= \left[{P}_{i}^{*}\text{log}{P}_{i}+(1-{P}_{i}^{*})\text{log}\left(1-{P}_{i}\right)\right]$$

where ${P}_{i}$, ${P}_{i}^{*}$ is the predicted class label, the actual class score (${P}_{i}$ is 1 if the anchor is positive and 0 if the anchor is negative), and ${t}_{i}$ ${t}_{i}^{*}$ are the predicted coordinates and actual coordinates.

The second fully connected layer regresses a four-dimensional vector representing the bounding box coordinates of the potential object within the proposal. This step utilizes a smooth L1 loss function^15^, as defined in Eqs. (3) and (4).

3 $${L}_{reg}\left({t}_{i},{t}_{i}^{*}\right)={\sum }_{i}{smooth}_{{L}_{i}}\left({t}_{i}-{t}_{i}^{*}\right)$$

4 $${smooth}_{{L}_{i}}\left(x\right)=\left\{\begin{array}{l}0.5 {x}^{2}\;\; if\, \left|x\right|<1\\ \left|x\right|\;\;-0.5\; otherwise\end{array}\right.$$

where ${t}_{i}$ is the regression parameter of the bounding box of the i-th anchor and ${t}_{i}^{*}$ is the regression parameter of the real box corresponding to the i-th anchor.

The fixed-size feature map from the backbone network and the Nx5 matrix from the RPN serve as inputs to the Region of Interest (ROI) layer. This layer extracts fixed-size feature maps for each proposal, contributing significantly to the training and testing efficiency of Faster R-CNN. Finally, each fixed-size ROI feature map is processed by a SoftMax layer for object classification and a fully connected layer for precise bounding box localization^21^. The overall loss function^15^ is defined in Eq. (5)

5 $$L\left(p,u,{t}^{u},v\right)={L}_{cls}\left(p,u\right)+\lambda \left[u\ge 1\right]{L}_{loc}\left({t}^{u},v\right)$$

where p is the SoftMax probability distribution predicted by the classifier, u is the actual class label of the target, v represents the bounding box regression parameters of the real target, and ${t}^{u}$ represents the regression parameters of the corresponding class u predicted by the bounding box regressor.

In this context, we utilized ResNeXt as the Faster RCNN backbone. ResNeXt offered the best compromise between accuracy and computational cost compared to alternatives like ResNet-50, DenseNet-121, EfficientNet-B0, MobileNetV2, and transformers. Despite, ResNeXt has more parameters (25M) than lightweight models (EfficientNet-B0: 5.3M, MobileNetV2: 3.4M, and DenseNet-121: 8M), its grouped convolutions enhance feature extraction without excessive computational cost, addressing the multi-class detection limitations of networks like EfficientNet-B0 and Although, DenseNet-121 improves feature reuse through dense connections, reducing redundancy and enhancing gradient flow, its densely connected structure can lead to increased memory consumption and computational complexity, making it less efficient for large-scale object detection compared to ResNeXt-50. Therefore, the bi-stage compression technique further refines ResNeXt-50’s efficiency, ensuring strong detection performance despite its parameter count^3^.

**Fig. 2:**
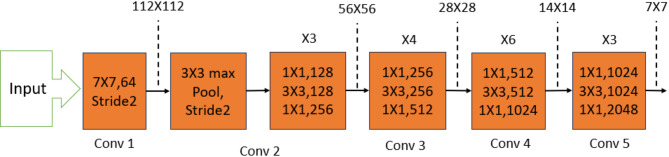
Resnext50_324d architecture.

ResNeXt, short for Aggregated Residual Transformations for Deep Neural Networks, builds upon the strengths of ResNet, VGG, and Inception architectures. It incorporates residual connections from ResNet, enabling the construction of deep models with repetitive layers similar to VGG. Additionally, ResNeXt leverages a “cardinality” parameter to control the number of parallel residual transforms within each block, addressing the high number of hyperparameters present in ResNet^16^. The specific architecture of ResNeXt is depicted in Fig. 2.

### Aware training

**Fig. 3:**
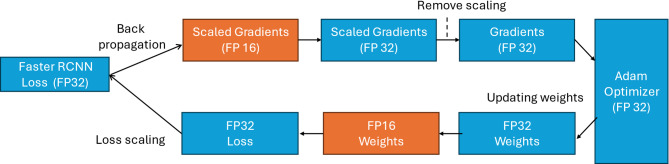
The employed mixed-precision training technique.

In the training phase, we employ mixed-precision training instead of the standard FP32 to encode model parameters, as well as to store weights, activations, and gradients. To preserve accuracy and address the limitations associated with FP16, we maintain a master copy of the weights in FP32. In each training iteration, during the forward pass of backpropagation, this master copy is converted to FP16. The resulting weight gradients are then utilized by the optimizer to update the master weights at the end of each iteration. This conversion from FP32 to FP16 effectively mitigates the precision loss that can occur with very small values, as FP16 supports a narrower range of representable values compared to FP32. This preservation of precision is crucial for maintaining the model’s accuracy. Furthermore, to address the issue of underflow, where gradients approach zero due to precision limitations, loss scaling is employed^17^. After the forward pass is completed and before backpropagation, the loss value is multiplied by a scale factor. This scale factor ensures that all gradients are subsequently scaled by the same factor, thereby bringing them within the range of FP16. Consequently, the master weights in FP32 are updated by dividing the gradients by the same scale factor. This process helps avoid underflow-related issues and ensures effective training^17^. Figure 3 illustrates the workflow and interactions involved in this approach.

### Post training

In post-training compression, we employ unstructured weight pruning to reduce model size and computational overhead, and dynamic quantization to convert weights and activations to lower precision formats, improving runtime efficiency and performance.

#### Pruning method

After completing Faster R-CNN model training, we employed the technique of global unstructured pruning, also referred to as weight pruning. This method, specifically utilizing the L1-Norm magnitude-based approach, involves assigning a zero value to the weight tensor’s least significant parameters to eliminate redundant parameters and sever connections between neurons. The primary objective of this pruning technique is to reduce the overall size of the Faster R-CNN model, enhance computational speed, and optimize storage efficiency, all while preserving the fundamental structure of the model intact.

#### Quantization method

Finally, Post-Training Quantization Method, specifically dynamic quantization is employed to reduce the precision of a model without any loss of information, efficient memory storage, and computational speed without performance reduction by compressing the data type of activation and weight tensors from 32-bit floating point (float32) to 8-bit integer (int8) but the weights are quantized in advance, while the activations are quantized dynamically during inference. The integration of the pruning method following the quantization method into the Faster R-CNN model contributes to the overall compression and optimization of the model, reduces memory requirements for storage, and improves computational speed with minimal performance loss.

## Experimental results

In this section, we performed experiments to assess the efficacy of Bi-Stage Compression, which combines pruning and quantization, for improving Faster R-CNN in satellite object detection. This process involved initially applying pruning to streamline the model by eliminating redundant parameters, followed by quantization to lower the precision of weights and activations. Subsequently, we evaluated the compressed model using a range of metrics on a diverse satellite imagery dataset.

### Datasets

The proposed method has been assessed on two commonly used datasets in the RS community: the NWPU VHR-10 dataset and the ship dataset.

**Fig. 4:**
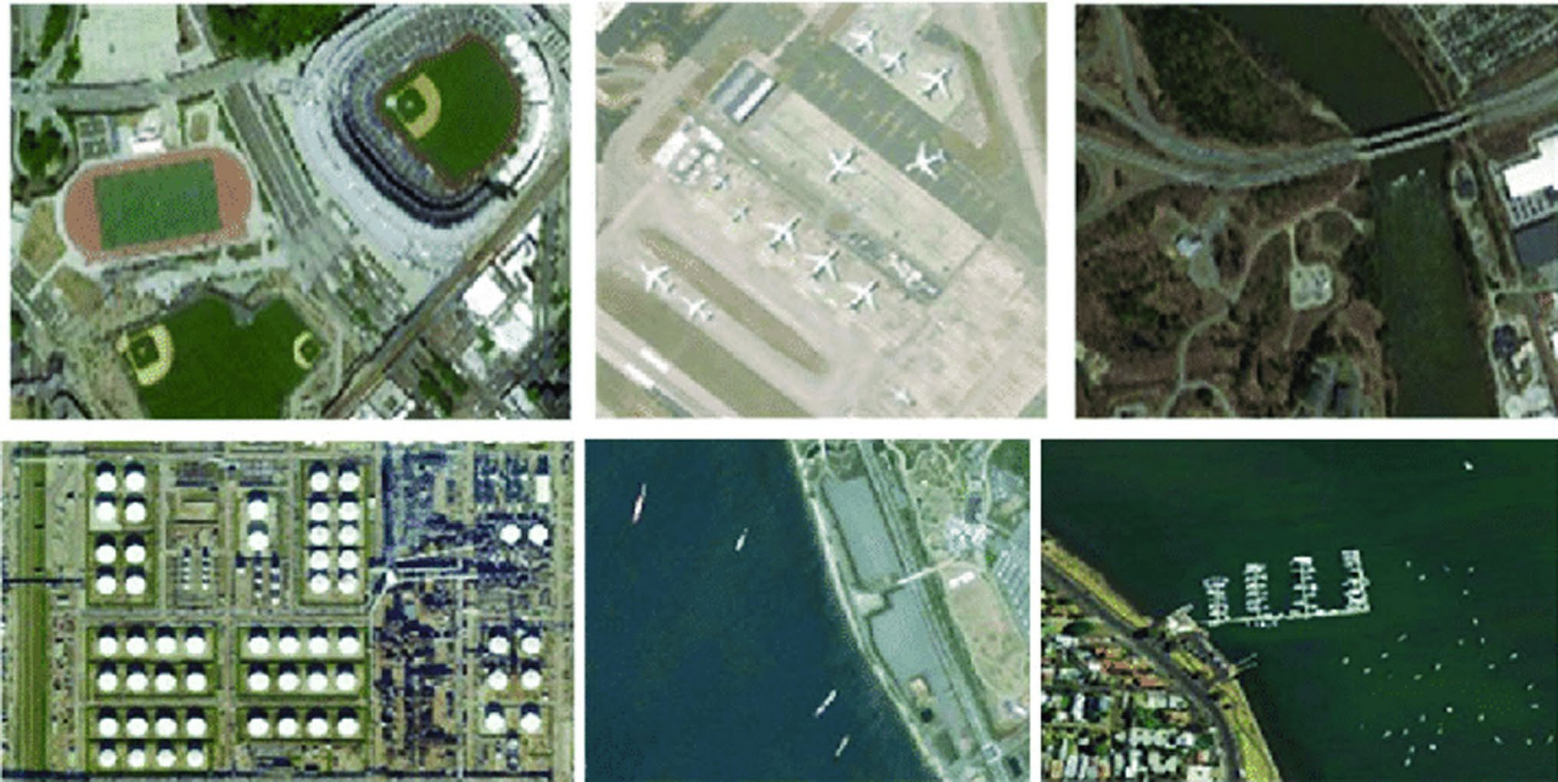
Samples of NWPUVHR-10 dataset.

NWPU VHR-10 dataset^18^ (accessed at https://www.kaggle.com/datasets/frinimoh) has 556 images that contain several airplane, ship, baseball diamond, tennis court, basketball court, ground track field, and vehicle items. The images are split into 80% training and 20% testing, with 440 samples in training and 2366 objects and 116 samples in testing and 1530 objects. Samples are displayed in Fig. 4.

**Fig. 5:**
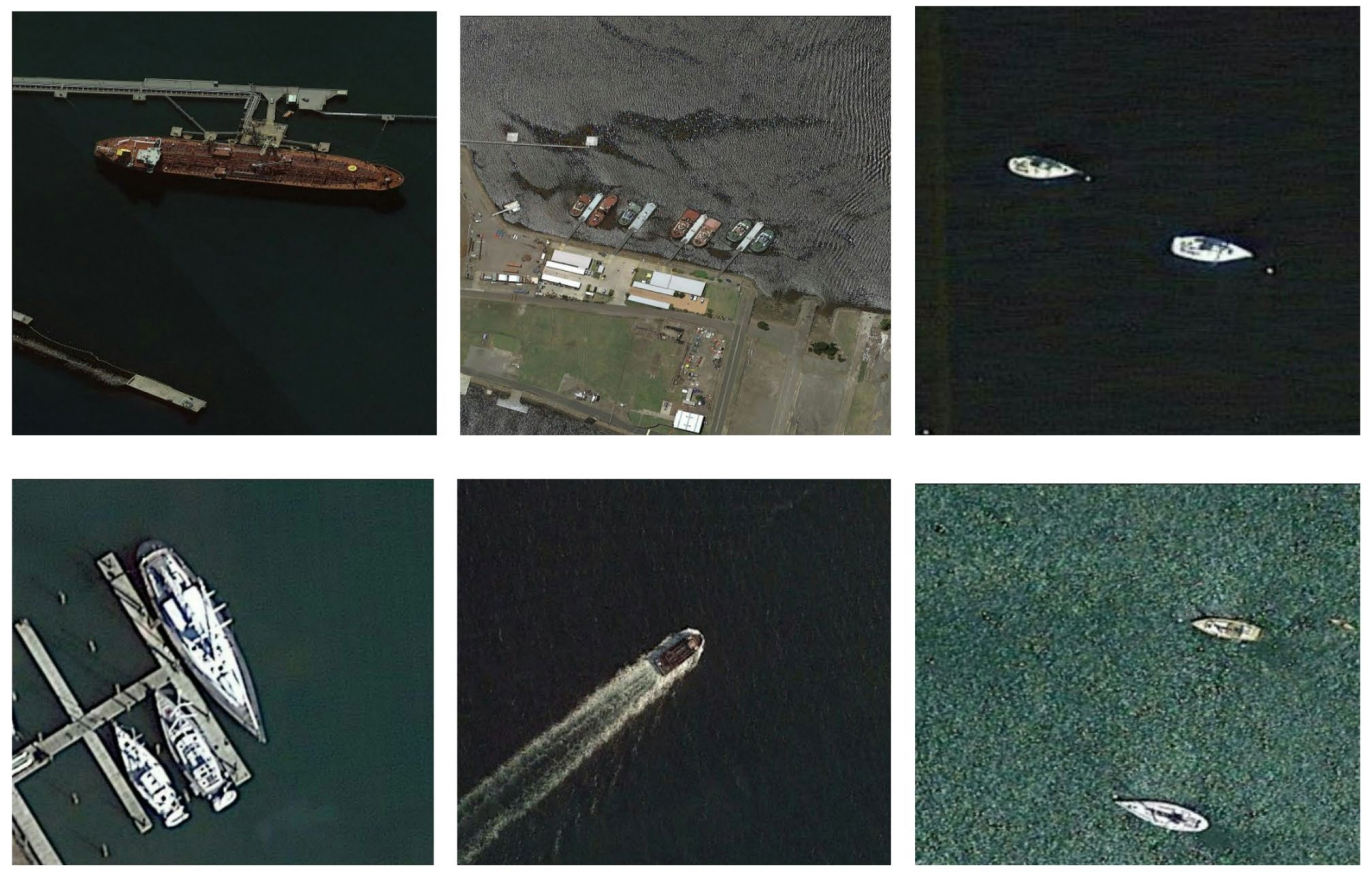
Samples of Ship dataset.

The Ship dataset^13^ comprises a total of 794 images obtained through extraction from Google Earth, with spatial resolution ranging from 20 to 50 cm. The images are split into 70%, 20% for validation, and 10% for testing, with 556 samples in training, in validation it is 159 samples, and in testing it is 79 samples. Subsequently, augmentation techniques were applied to the training images, specifically horizontal flipping, and a 2× rotation which resulted in an increase in images to 1400 images and an increase in the number of ship dataset images to become 1638 images. To standardize the dataset, all images were resized to dimensions of 640 × 640, and annotations were transformed from Pascal VOC to YOLOv5/8 format. as shown in Fig. 5.

### Experimental setup

To ensure fair and unbiased competition, the proposed method has been trained for 30, 50, 70, and 100 epochs with a stochastic gradient descent (SGD) optimizer, adopting a learning rate (LR) equal to 0.01, momentum equal to 0.9, batch size equal to 16, and weight decay equal to 0.0005. Training and inference are performed on a Tesla T4 (GPU) machine by Google Colab with GPU RAM of 16 GB, system RAM of 13 GB, and disk space of 80 GB. Furthermore, to enhance training efficiency, a mixed-precision training technique was implemented using the automatic mixed precision (AMP) functionality. This was facilitated by Apex, an extension for PyTorch developed by NVIDIA^22^.

Regarding Yolo8n and Yolo8m, we utilized a stochastic gradient descent (SGD) optimizer, adopting a learning rate (LR) equal to 0.01, momentum equal to 0.9, batch size equal to 16, and weight decay equal to 0.0005. Training and inference are performed on a Tesla T4 (GPU) machine by Google Colab with GPU RAM of 16 GB, system RAM of 13 GB, and disk space of 80 GB.

### Evaluation metrics

The effectiveness of the proposed approach was evaluated using various metrics, including Precision (P), Recall (R), F1-score, average precision (AP), and mean average precision (mAP). These metrics were defined by Eqs. (6), (7), (8)^17^, (9), and (10)^20^, respectively. Furthermore, the proposed approach has also been assessed in terms of the number of parameters, model size, and training duration.

6 $$P=\frac{TP}{TP+FP}$$

7 $$R=\frac{TP}{TP+FN}$$

8 $$F1-score=2\frac{Precision*Recall}{Precision and Recall}$$

9 $$AP=\frac{1}{11}*Sum\left(11\; point\; interpolated\; precision\right)$$

10 $$mAP=\frac{1}{n}*sum\left(AP\right)$$

where TP is the True Positives (correctly predicted positives), FP is the False Positives (incorrectly predicted positives), and FN is the False Negatives (incorrectly predicted negatives).

### Results

In this section, we conducted several experiment sets to evaluate the performance of the proposed approach in terms of precision, recall, F1-score, average precision (AP), number of parameters of the model, the model size, and the time of the training.

#### The NWPU VHR-10 dataset

**Fig. 6:**
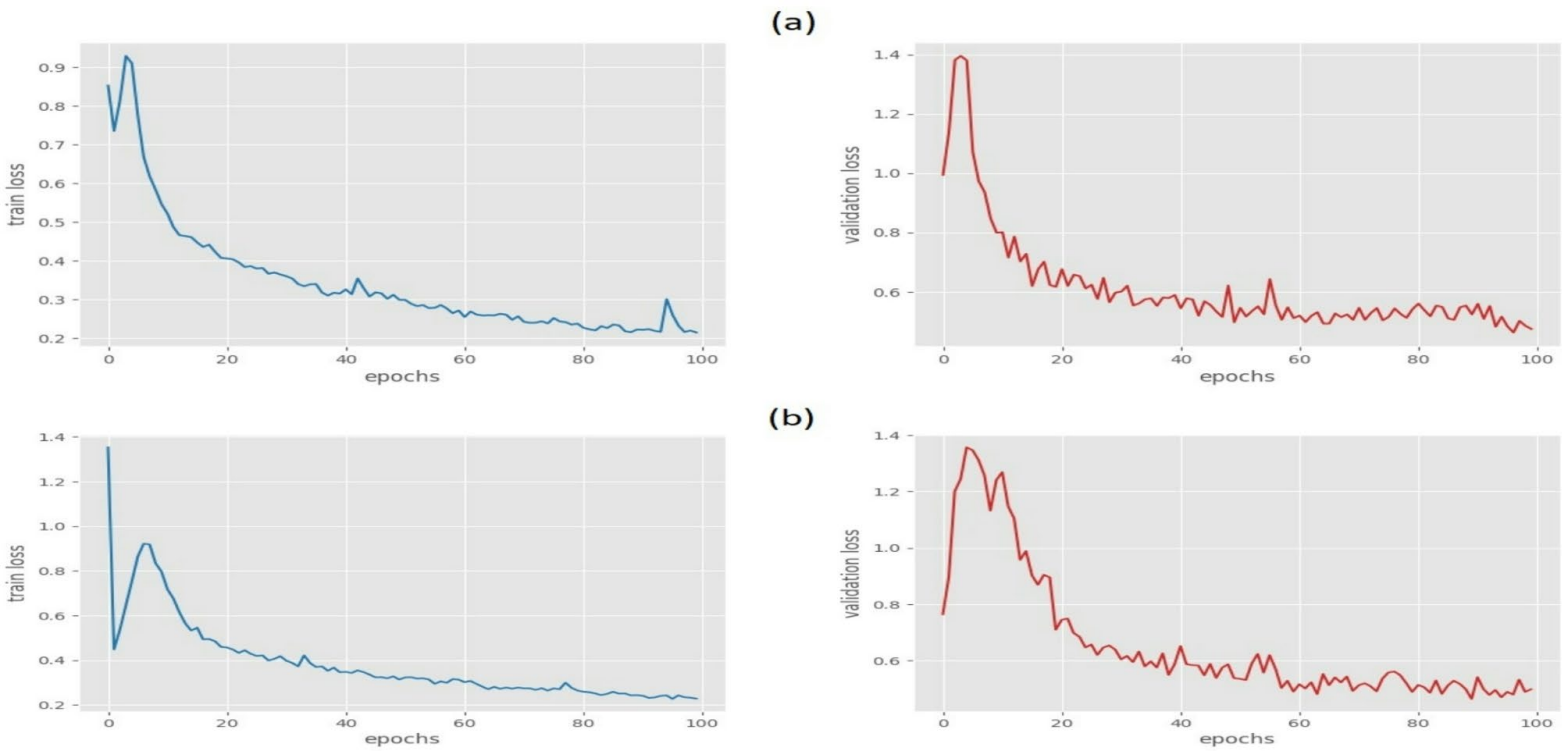
The training and validation loss (**a**) Faster-RCNN without Mixed Precision training, (**b**) Faster-RCNN with mixed precision training.

First, Fig. 6 depicts the training and validation loss for Faster-RCNN with and without mixed precision training.1. One can observe that the mean mAP with mixed precision training is slightly lower than that without mixed precision training, but the difference ranges was between 0.1 to 0.4. The training time was reduced from 37.7% to 44.4% across different epochs. For example, at epoch 100, mixed precision training takes only 57% of the time as training without mixed precision, and at epoch 70, mixed precision training takes only 56% of the time as training without mixed precision. Analytically, the reduction in training time hit about 33.6% with a small trade-off in accuracy, typically around 0.1% to 0.4%. One can argue that this negligible variation hardly justifies the significant increase in training time.

Next, Table 2 compares the performance of different configurations of Faster-RCNN models trained with mixed precision (MP), quantization (Q), and unstructured pruning (UNP) in terms of precision, recall, F1-score, mAP, size (MB), and the number of parameters. All configuration settings achieved similar detection accuracy around 89.4 in terms of mAP, but mixed precision training with UNP achieved a significant reduction in parameter count (approximately 23%) compared to the baseline (40–41 million parameters). Additionally, all models with Q achieve a smaller size (approximately 26% reduction) compared to the baseline (164 MB). It is noteworthy that combining Q and UNP leads to reduced size (approximately 26% reduction) and parameters (approximately 57% reduction) compared to the baseline with a slight improvement in AP (from 89.43 to 89.53). The proposed approach achieves the smallest model size (122 MB) and number of parameters (17.7 million), it comes with a slight increase in mAP, which is most likely attributed to the application of pruning.

**Table 2:** Comparison of efficiency gains in faster-RCNN with mixed precision training and model compression techniques.

Model	Recall	Precision	F1-Score	AP	mAP	Size (MB)	# Parameters
Faster R-CNN	91.50	97.50	93.00	89.63	89.6	164	40791804
Faster-R-CNN + MP	91.31	97.28	92.81	89.43	89.4	164	40791804
Faster-R-CNN + MP + Q	91.31	97.19	92.53	89.38	89.4	122	26849999
Faster-R-CNN + MP + UNP	91.36	97.06	92.84	89.52	89.5	164	31627132
Faster-R-CNN + MP + UNP + Q (proposed)	91.36	97.10	92.84	89.53	89.5	122	17685327

**Fig. 7:**
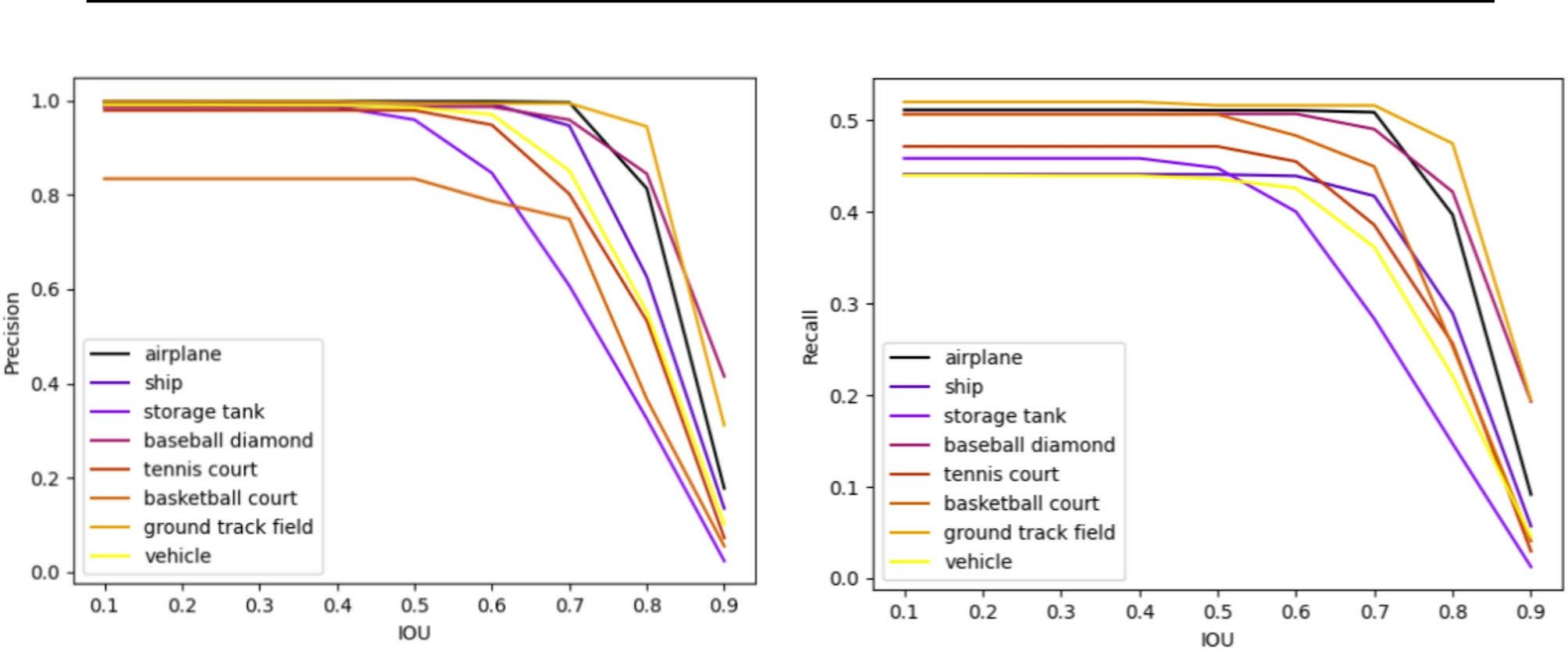
Precision-recall curve of the proposed model in airplane, ship, baseball diamond, tennis court, basketball court, ground track field, and vehicle classes.

Next, Table 3 compares the precision (P), recall (R), F1-score, and average precision (AP) of the proposed bi-stage compression approach of Faster-RCNN. As can be seen, the model achieves high overall performance across most classes, particularly excelling in detecting objects such as airplanes, baseball diamonds, and ground track fields, with scores exceeding 95% in Recall, Precision, F1-Score, and Average Precision. However, while the model demonstrates strong performance overall, it shows relatively lower precision in classes like basketball courts and lower recall in classes like tennis courts, and vehicles. Performance varies slightly between classes, with “Airplane” achieving the highest precision scores of around 99%, whereas “Basketball court” exhibits the lowest precision at 83.4%. The trade-off between precision and recall for the proposed method model in each class at eight different IoU (Intersection over Union) thresholds from 0.1 to 0.9 to show the performance of the proposed model is depicted in Fig. 7.

**Table 3:** Performance of the proposed bi-stage compression for faster R-CNN in terms of precision (P) and recall (R). F1-Score, and average precision (AP) for each class.

Classes	Recall	Precision	F1-Score	AP
Airplane	99.5	99.9	99.0	99.4
Baseball diamond	96.3	98.7	95.8	95.3
Basketball court	91.7	83.4	86.6	81.2
Ground track field	95.7	99.8	97.8	95.7
Ship	83.8	99.5	89.1	83.5
Storage tank	90.7	98.6	94.1	89.9
Tennis court	90.1	97.9	92.3	88.7
Vehicle	83.1	99.0	88.0	82.5

**Fig. 8:**
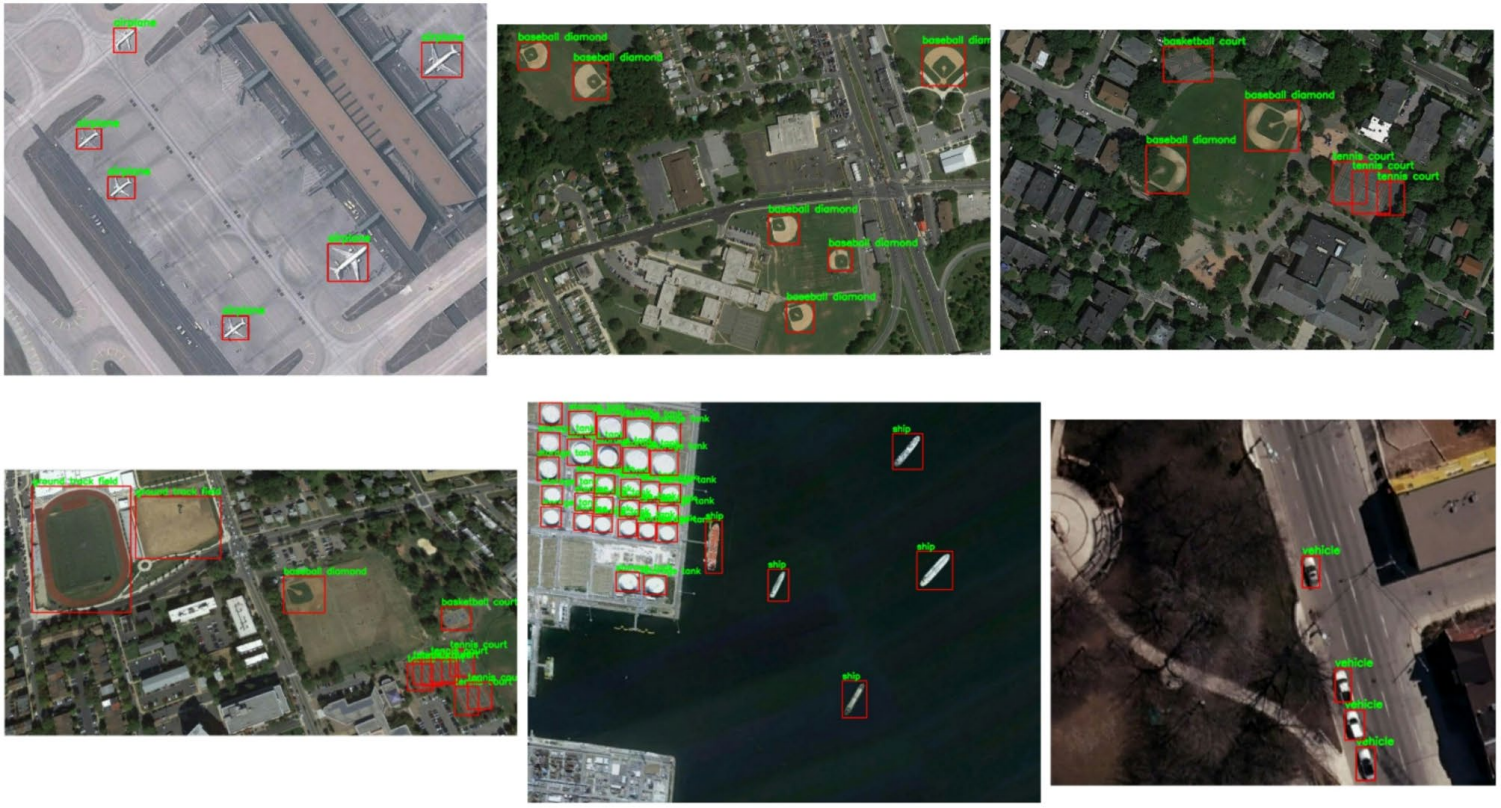
Samples of the results of the proposed method for the NWPU VHR-10 dataset.

Next, it can be observed that the airplane and ground track field consistently exhibit high recall and precision scores across all IOU thresholds, showcasing robust object detection capabilities. Ships also maintain strong performance, while storage tank, baseball diamond, and vehicle show some variability in performance, with decreasing scores at higher IOU thresholds. Basketball court and tennis court demonstrate sensitivity to IOU thresholds, with significant drops in performance as the threshold increases. Overall, despite variations in sensitivity to IOU thresholds, all classes generally achieve relatively high recall and precision scores, suggesting effective object detection capabilities with some classes being more sensitive to threshold variations than others. Overall, although some classes may show greater sensitivity to threshold changes than others, all classes generally achieve high recall and precision scores. Samples of the results of the proposed method are shown in Fig. 8.

Finally, in comparison to other YOLO models, Table 4 shows that our Bi-Stage Compression technique for Faster R-CNN achieves the maximum precision (97.10%), recall (91.36%), F1-score (92.84%), and mAP (89.50%), demonstrating greater accuracy. Nevertheless, this results in a higher model size (122MB, 17.6M parameters) and a longer training period (1.8 h). On the other hand, YOLO models, especially YOLOv8n (0.210 h, 12MB, 3M parameters), emphasize efficiency and speed, which makes them ideal for real-time applications. With an F1-score of 92.69%, YOLOv11m outperforms the other YOLO versions; nevertheless, it still falls short of our suggested approach in mAP (66.15%).

**Table 4:** Comparison of efficiency, time (Hr), size (MB), and number of parameter gains in the proposed bi-stage compression for faster R-CNN, and different versions of Yolo.

Model	Time (H)	Parameters	Size (MB)	Precision (%)	Recall (%)	F1-score (%)	MAP (%)
YOLOv8n	0.210	3,012,396	12	91.93	67.63	77.93	59.94
YOLOv8m	0.382	25,860,850	10	94.02	72.20	81.68	65.22
YOLOv10m	0.436	16,493,244	66	92.96	69.20	79.34	64.89
YOLOv11m	0.418	20,059,082	81	94.99	75.72	84.27	66.15
Proposed approach	1.8	17,685,327	122	97.10	91.36	92.84	89.50

#### The ship dataset

**Fig. 9:**
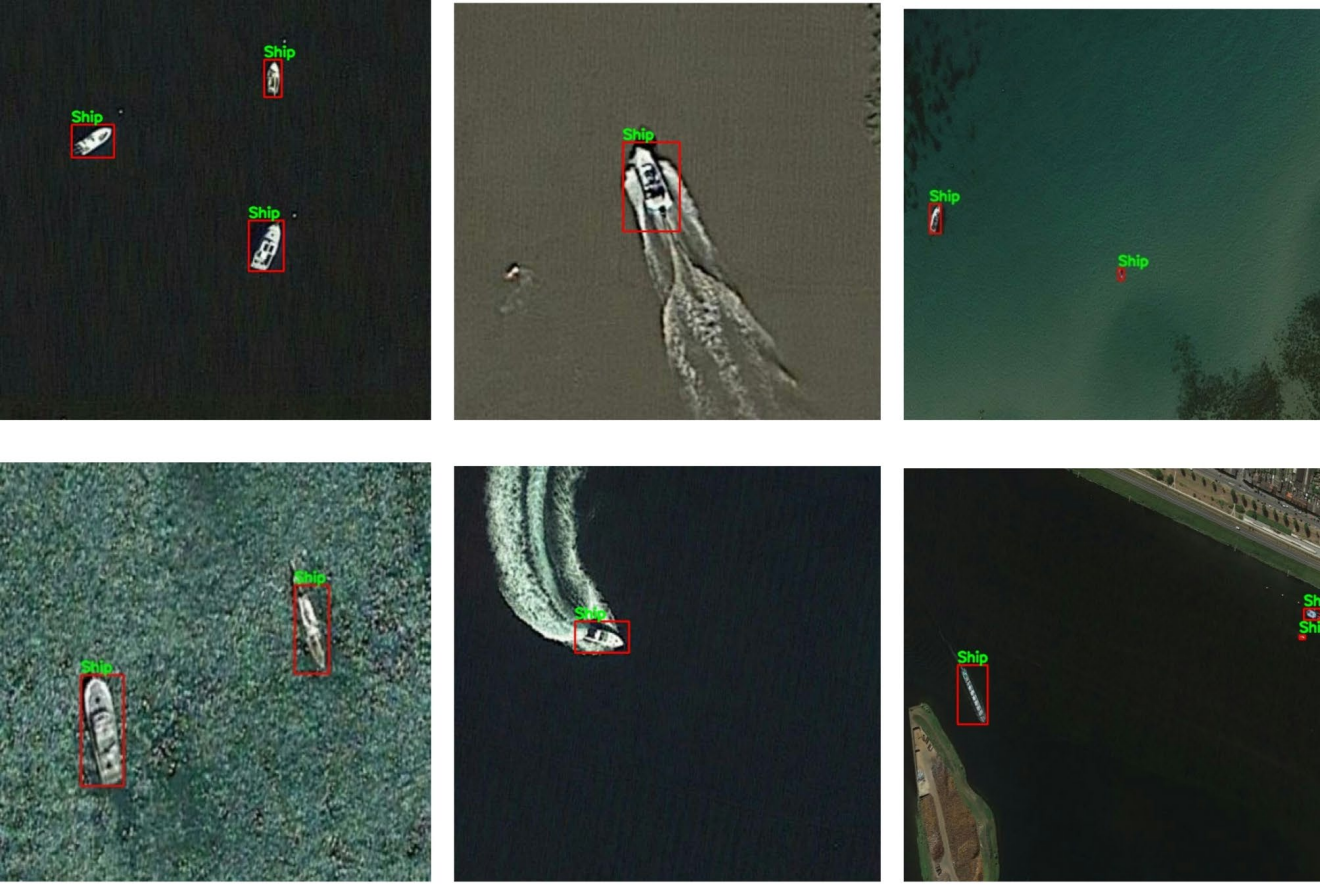
Samples of the obtained results of the proposed method for the Ship dataset.

The behavior of Faster-RCNN, the proposed Faster-RCNN with mixed precision training, Yolo8n, and Yolo8m have been compared in terms of efficiency techniques and time (HR) using the ship dataset. Table 5 compares the efficiency and accuracy of four object detection models: Faster-RCNN, Faster-RCNN with Mixed Precision training (MP), Yolo8n, Yolo8m, Yolo10m, and Yolo11m. It evaluates them using metrics like recall, precision, average precision, mean average precision, and time (in hours). As you can see, Faster-RCNN achieves the highest accuracy (96.4% recall, 99.0% precision, and 95.9% mAP) but has the slowest training time (3 h). Faster-RCNN with MP, a variant of Faster-RCNN, achieved a slightly lower mAP of 95.4% but offers a significant speedup (1.8 h) in training time. While YOLO8m marginally increases accuracy (71.0% mAP) with a training duration of 0.464 h, YOLO8n prioritizes speed with the smallest training time (0.283 h) at the expense of accuracy (68.6% mAP). While YOLO11m achieves a high precision (99.7%) but lower overall accuracy (67.4% mAP) with a training time of 1.142 h, the recently updated YOLO10m model strikes a compromise between efficiency and accuracy (72.5% mAP in 1.128 h). Samples of the results from the proposed method are shown in Fig. 9.

**Table 5:** Comparison of efficiency and time (Hr) gains in faster-RCNN without and with mixed precision training (MP), Yolo8n and Yolo8m.

Model	Recall	Precision	F1-Score	AP	mAP	Time (Hr)
Faster-RCNN	96.4	99.0	95.0	95.86	95.9	3
Yolo8n	70.3	96.7	77.9	68.56	68.6	0.283
Yolo8m	73.0	96.8	81.4	71.02	71.0	0.464
Yolo10m	73.0	99.3	83.1	72.51	72.5	1.128
Yolo11m	67.6	99.7	80.2	67.37	67.4	1.142
Proposed approach	95.5	99.1	96.3	95.37	95.4	1.8

## Discussion

The above experiments demonstrate that different configurations of Faster R-CNN models, trained using the proposed bi-stage compression approach on the NWPU VHR-10 dataset, attain similar levels of detection accuracy, with an average mAP (mean Average Precision) of approximately 89.4%. with a reduction of model size by approximately 26% and a parameter count of approximately 57% compared to the baseline model. Additionally, it demonstrates a slight improvement in average precision (AP) from 89.43 to 89.53. However, when contrasted with YOLO models, especially variants like YOLOv8n, they are optimized for speed and minimal model size. While they demonstrate respectable F1-scores, their mAP values are notably lower than those obtained by our proposed method. We also tested our method on the ship dataset. While the compressed Faster R-CNN model outperformed YOLO8n, YOLO8m, YOLO10m, and YOLO11m in terms of accuracy (mAP of 95.4% vs. 68.6% vs. 71.02% vs. 72.5% and 67.37, respectively), it required a longer training time (1.8 h vs. 0.283 vs. 0.464 vs. 1.128, and 1.142 h).

Despite its potential, the proposed bi-stage compression approach presents several inherent challenges. Mixed-precision training (FP16) introduces numerical instability, particularly in deep networks, necessitating complex loss scaling and potentially impacting convergence. Reduced gradient fidelity from FP16 and the risk of critical parameter elimination via unstructured pruning can degrade accuracy. Furthermore, dynamic quantization may compromise model robustness, especially in high-precision tasks. Addressing these limitations requires rigorous hyperparameter optimization and the exploration of adaptive pruning, gradient scaling, and hybrid quantization techniques to ensure robust and efficient model compression.

In conclusion, our bi-stage compression approach, combining mixed-precision training, unstructured weight pruning, and dynamic quantization, offers a practical solution for deploying Faster R-CNN models in remote sensing applications. It significantly reduces model size and parameter count while maintaining high detection accuracy.

## Conclusion

Object detection approaches play a crucial role in various remote sensing applications, including urban detection, precision farming, and disaster prediction. While Faster R-CNN has become popular for its high performance, it imposes significant computational, and storage demands. To address these challenges, this study introduces a novel hybrid compression approach for Faster R-CNN, specifically tailored for the remote sensing domain. The proposed bi-stage compression strategy begins with aware training using mixed-precision FP16, which reduces training time by 1.5 × to 5.5 × without affecting performance or memory usage. In the post-training phase, unstructured weight pruning is employed to reduce the number of parameters, followed by dynamic quantization to further minimize network size during runtime. The effectiveness of this approach is demonstrated through evaluations on the NWPU VHR-10 and Ship datasets, which show an average 25.6% reduction in model size and a 56.6% reduction in parameters, all while maintaining the mean Average Precision (mAP). The proposed approach offers a promising solution for deploying high-performance object detection models in resource-constrained remote sensing applications. In the future, we will adopt loss scaling for numerical stability, advanced gradient management to prevent under/overflow, and hybrid precision schemes for optimal speed/accuracy balance in mixed-precision training.

## Data Availability

The datasets used and/or analyzed during the current study are available from the corresponding author upon reasonable request.
